# Norlobaridone Inhibits Quorum Sensing-Dependent Biofilm Formation and Some Virulence Factors in *Pseudomonas aeruginosa* by Disrupting Its Transcriptional Activator Protein LasR Dimerization

**DOI:** 10.3390/biom13111573

**Published:** 2023-10-24

**Authors:** Raya Soltane, Ahlam Alasiri, Mostafa N. Taha, Rehab H. Abd El-Aleam, Kawthar Saad Alghamdi, Mosad A. Ghareeb, Doaa El-Ghareeb Keshek, Susana M. Cardoso, Ahmed M. Sayed

**Affiliations:** 1 Department of Basic Sciences, Adham University College, Umm Al-Qura University, Makkah 21955, Saudi Arabia; ajasiri@uqu.edu.sa; 2 Microbiology and Immunology Department, Faculty of Pharmacy, Nahda University, Beni-Suef 62764, Egypt; moustafa.nasr@nub.edu.eg; 3 Pharmaceutical Chemistry Department, Faculty of Pharmacy, Modern University for Technology and Information (MTI), Cairo 11571, Egypt; rehab.hamed@pharm.mti.edu.eg; 4 Department of Biology, College of Science, University of Hafr Al Batin, Hafar Al Batin 39511, Saudi Arabia; ksalghamdi@uhb.edu.sa; 5 Medicinal Chemistry Department, Theodor Bilharz Research Institute Kornaish El Nile, Warrak El-Hadar, Imbaba, P.O. Box 30, Giza 12411, Egypt; m.ghareeb@tbri.gov.eg; 6 Department of Biology, Jumum College University, Umm Al-Qura University, Makkah 21955, Saudi Arabia; dekeshek@uqu.edu.sa; 7 Agriculture Genetic Engineering Research Institute (AGERI), Agriculture Research Center, Giza 11571, Egypt; 8 LAQV-REQUIMTE, Department of Chemistry, University of Aveiro, 3810-193 Aveiro, Portugal; susanacardoso@ua.pt; 9 Pharmacognosy Department, Faculty of Pharmacy, Nahda University, Beni-Suef 62513, Egypt

**Keywords:** quorum sensing, *Pseudomonas aeruginosa*, LuxR-type receptors assay, LasR, thermal shift assay, MD simulation

## Abstract

In the present study, norlobaridone (NBD) was isolated from *Parmotrema* and then evaluated as a new potent quorum sensing (QS) inhibitor against *Pseudomonas aeruginosa* biofilm development. This phenolic natural product was found to reduce *P. aeruginosa* biofilm formation (64.6% inhibition) and its related virulence factors, such as pyocyanin and rhamnolipids (% inhibition = 61.1% and 55%, respectively). In vitro assays inhibitory effects against a number of known LuxR-type receptors revealed that NBD was able to specifically block *P. aeruginosa*’s LasR in a dose-dependent manner. Further molecular studies (e.g., sedimentation velocity and thermal shift assays) demonstrated that NBD destabilized LasR upon binding and damaged its functional quaternary structure (i.e., the functional dimeric form). The use of modelling and molecular dynamics (MD) simulations also allowed us to further understand its interaction with LasR, and how this can disrupt its dimeric form. Finally, our findings show that NBD is a powerful and specific LasR antagonist that should be widely employed as a chemical probe in QS of *P. aeruginosa*, providing new insights into LasR antagonism processes. The new discoveries shed light on the mysterious world of LuxR-type QS in this key opportunistic pathogen.

## 1. Introduction

The overuse of antibiotics in conventional therapy for the treatment of bacterial illnesses has led to multidrug-resistant organisms that endanger human health and the environment [1,2]. In recent years, efforts have been made to enhance public awareness of antimicrobial resistance (AMR) and the serious challenges it poses to modern health standards [3].

*Pseudomonas aeruginosa* is a Gram-negative bacterium that causes acute and chronic infections in immunocompromised people, including cystic fibrosis and burns [4,5]. The growing number of bacteria resistant to numerous medicines makes typical antibiotic-induced treatment extremely difficult [6,7,8,9,10]. As a result, new ways of fighting infections caused by this infamous disease are urgently needed [11,12,13]. *P. aeruginosa*’s ability to cause disease is dependent on the development of chemicals known as “virulence factors” that actively harm host tissue [11,14,15,16]. A promising novel treatment method for treating *P. aeruginosa* infections has been found by targeting virulence factors (e.g., reducing their synthesis). In general, this reduces the bacterium’s pathogenicity and enhances the possibility that the host’s immune system will be able to eradicate the infection before it causes significant tissue damage [11,15,17,18,19]. *P. aeruginosa* produces a variety of virulence factors, including biofilm components, pyocyanin, and rhamnolipids [20].

**Figure 1:**
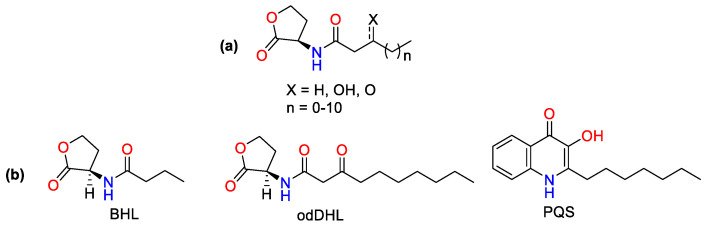
(**a**) AI-1/AHL, (**b**) Respective AIs of *las*, *rhl* and PQS systems which regulate the transcription of virulence factors: The *N*-butanoyl-L-homoserine lactone (BHL), the *N*-(3-oxododecanoyl)-L-homoserine lactone (odDHL) and the 2-heptyl-3-hydroxy-4-quinolone (PQS).

**Figure 2:**
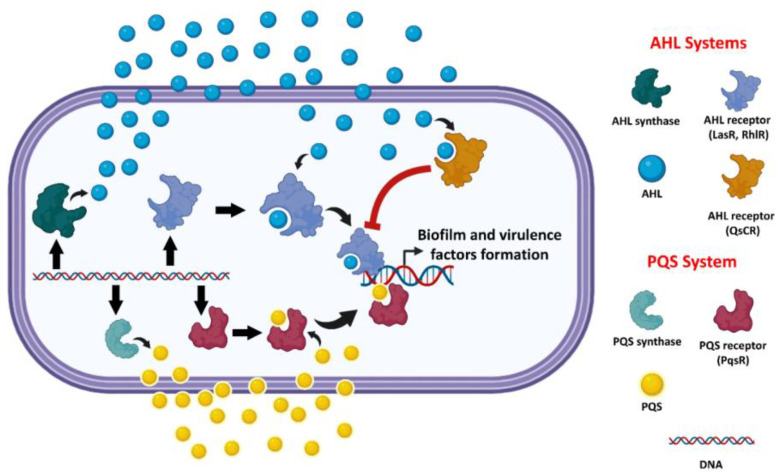
A general schematic illustration of *P. aeruginosa* AHL-type and PQS-type QS systems.

*P. aeruginosa’s* virulence factor production is controlled by an intercellular signaling pathway known as quorum sensing (QS) [21,22]. Many bacterial species employ QS systems, which enable coordinated interactions among cells in a population [17]. Small diffusible signaling molecules known as autoinducers (AIs) mediate this communication pathway [17,23,24]. N-acylated L-homoserine (AHL, Figure 1a) is a self-inducing chemical in most Gram-negative bacteria [23,24]. They are generated by LuxI-type synthase enzymes and bind to LuxR-type cytoplasmic receptors to induce gene expression in a bacterial population [11,23,24,25,26]. In general, each bacterial species responds to its own AHL differently, employing various LuxI-type synthases and LuxR-type receptors [24,27]. *P. aeruginosa* has two AHL-based QS systems: the first is N-butanoyl-L-homoserine lactone (BHL, Figure 1b), which is produced by RhlI and recognized by RhlR, and the second is N-3-oxododecanoyl-L-homoserine lactone (OdDHL, Figure 1b), which is produced by LasI and detected by LasR. Furthermore, there is a third QS system that employs a different type of receptor (a LysR-type receptor known as PqsR) and a chemically unique autoinducer (PQS) (Figure 1b and Figure 2). The PQS system is linked to the two AHL-based systems, forming a complex hierarchical QS network that regulates various aspects of *P. aeruginosa’s* pathogenicity [11,24,28,29]. The final system (QscR) is a LuxR-type receptor that lacks a related synthase and native AHL autoinducer but can be triggered by OdDHL. QscR acts as a regulator for LasR and RhlR, repressing their activity in response to high odDHL production (Figure 2).

Considering the essential role of quorum sensing in virulence, perturbing bacterial quorum sensing pathways, also known as quorum quenching (QQ), has been proposed as a viable strategy to combat the dangerous problem of bacterial resistance to antibiotics [30,31,32,33,34,35,36,37]. This idea is founded on the fact that antibiotics target essential bacterial cellular processes, thereby stimulating the evolution of resistant strains. Since QS is not required for bacterial survival and is primarily used for virulence control, disruption of the QS pathways may impose less evolutionary pressure on bacteria to develop resistant strains, resulting in an effective antibacterial strategy [38,39].

Antibacterial drugs have been investigated to target *P. aeruginosa* QS systems, either by blocking the autoinducers’ synthase enzymes, hydrolyzing synthesized autoinducers, or antagonizing the autoinducers’ receptors (Figure 2).

**Figure 3:**
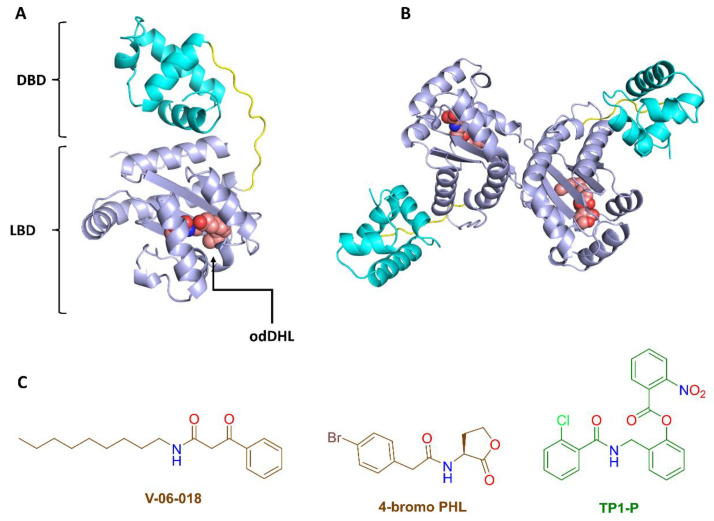
(**A**,**B**) Tertiary and quaternary LasR structures. The tertiary structure of LasR is made up of two domains: (i) the ligand binding domain (LBD) and (ii) the DNA binding domain (DBD). The LBD contains a conserved hydrophobic cavity for the native agonist odDHL. The functioning LasR form is the dimeric one (i.e., the quaternary structure). (**C**) Examples of previously reported LasR antagonists (red structures) and agonists (green structure).

LasR’s structure has been well characterized, and it is regarded to be the most attractive target for developing effective antagonists. A number of synthetic LasR agonists and antagonists have recently been produced (Figure 3C) [33,40]. As the LasR odDH-binding pocket is very flexible and can be occupied by a diverse variety of ligands that can function as either agonists or antagonists, it has been difficult to design effective, competitive LasR antagonists. Native LasR agonists (e.g., odDHL) or synthetic agonists (e.g., TP1-P; Figure 3C) are required for folding LasR into a stable tertiary structure (Figure 3A), which then oligomerizes into a functional dimeric quaternary structure capable of binding to DNA and acting as a transcription factor (Figure 3B) [33,40].

Lichens are a fungus that have adapted to have a symbiotic relationship with algae or cyanobacteria and require their specific nutrients to thrive. Several species of lichen have a long history of usage in traditional medicine and are currently enjoying a surge of popularity as an alternative treatment for a wide range of medical conditions around the world.

In addition to the multiple pharmacological activities of lichens, they have also been frequently reported for their antibacterial and antibiofilm potential [41,42,43,44].

The genus *Parmotrema* is one of the widespread lichens that have been used in many countries as an excellent source of multivitamins and bioactive phytochemicals [45]. In addition, several *Parmotrema* species have also been reported for their broad spectrum antibiofilm and antibacterial potential [46,47].

The primary objective of this study is to characterize a novel, potent QS inhibitor capable of preventing *P. aeruginosa* biofilm formation. We have found that one of our in-house crude extracts (i.e., *P. tinctorum* extract) exhibits interesting antibiofilm inhibition. Following bioactivity-guided chromatographic isolation, the most powerful antibiofilm candidate was submitted to a series of in vitro experiments to determine its mechanism of action. The exact mechanism of action was explained using computer-based modelling and molecular dynamics (MD) simulations. We hope that the results presented in this study will lead to the discovery of a new generation of potent QS inhibitors to be used against *P. aeruginosa*.

## 2. Materials and Methods

### 2.1. Chemicals and Buffers

The plasmids and bacterial strains used in this study are detailed in Table S1. Autoinducers (i.e., odDHL and BHL) were acquired from Sigma Aldrich. For the initial antibacterial and antibiofilm evaluation, isolated NBD (from our in-house natural products library; purity 90%) was used, whereas purchased NBD (purity ~95%; BOC Sciences™) was utilized for the remaining in vitro experiments. The structure of NBD was confirmed by comparing its ^1^H NMR and ^13^C NMR data (Figures S7–S9) with those previously reported [48]. The bacteria were grown in Luria–Bertani (LB) medium at 37 °C. The absorbance at 600 nm (OD_600_) was used to determine the bacterial growth rate. The absorbance and fluorescence of the samples were monitored using a Biotek Synergy 2 plate reader. Z buffer (60 mM Na_2_HPO_4_ + 40 mM NaH_2_PO_4_ + 10 mM KCl), phosphate buffer (60 mM Na_2_HPO_4_ + 40 mM NaH_2_PO_4_), phosphate buffered saline (137 mM NaCl + 2.68 mmol KCl, 10 mM Na_2_HPO_4_ + 1.8 mM KH_2_PO_4_), buffer A (20 mM TrisHCl, pH 8 + 1 mM DTT), buffer B (1 M NaCl, pH 8), and thermal shift assay reaction buffer (20 mM Tris-HCl, pH 8, 200 mM NaCl, and 1 mM DTT) were all utilized in various in vitro biological investigations. The dose–response curves were generated using GraphPad Prism (version 8). All biological investigations are described in detail in the supplementary file.

### 2.2. Growth Curve Analysis

The effect of NBD sub-inhibitory concentration on *P. aeruginosa* growth was investigated using a growth curve study. A total of 100 mL of LB broth supplemented with 5 μg/mL of the tested substance, and phosphate saline buffer as a negative control, were administered to overnight cultures of *P. aeruginosa*. The flasks’ OD_600_ was measured at 1 h intervals for up to 24 h at 37 °C [49].

### 2.3. Evaluation of the Inhibitory Effect on the Formation of Virulence Factors

#### 2.3.1. Inhibitory Effect on the Biofilm Formation

In 96-well polystyrene plates, the influence of NBD on biofilm growth was studied. The experiment was carried out in the manner reported by Stepanovi et al. [50], with a few minor changes. *P. aeruginosa* cells were cultured in LB medium, vortexed, then diluted 1:100 in new LB media to a final concentration of 1.5 × 108 CFU/mL (0.5 McFarland) for biofilm development. Cells were then cultured for 24 h at 37 °C with or without NBD. Following that, non-adherent bacteria were washed away in sterile phosphate-buffered saline (PBS), and adherent bacteria were stained for 15 min with a 1% crystal violet solution. Water was used to clean the wells in order to remove any remaining stains. The absorbance of the crystal violet solution at OD_570_ was determined by spectrophotometric analysis. Wells with phosphate buffer-enriched media were utilized as negative controls. As a positive control, azithromycin (AZM) was used, which inhibits biofilm formation at sub-inhibitory concentrations [51]. The percentage of inhibition was estimated using the following equation [52]:

$$\%\;\mathrm{of}\;\mathrm{inhibition}=\frac{\mathrm{Control}\;\mathrm{OD}-\mathrm{Test}\;\mathrm{OD}}{\mathrm{Control}\;\mathrm{OD}}\times 100$$

#### 2.3.2. Inhibitory Effect on Pyocyanin Production

Essar and coworkers’ (1990) [53] chloroform-HCl extraction was employed to measure pyocyanin production with minor changes. In brief, 7.5 mL of *P. aeruginosa* culture supernatant that had been exposed to NBD or not was extracted using 4.5 mL of chloroform and 1 mL of HCl (0.2M). The solution’s absorbance was measured at 520 nm. The pyocyanin concentration was calculated by multiplying the absorbance by 17.072. A sub-MIC concentration of AZM served as the positive control.

#### 2.3.3. Inhibitory Effect on Rhamnolipids Production

According to Abdel-Mawgoud and colleagues [54], the bacterial cultures were grown in a low salt medium for 48 h at 37 °C with 180 rpm shaking. This technique allowed us to assess the effects of NBD and the sub-MIC of AZM on rhamnolipid production. A total of 500 μL of culture supernatant was extracted with 3 mL of diethyl ether. The ether fractions were reconstituted in 500 μL of phosphate buffer (pH 8.0) after being collected, mixed, and dried in an 80 °C water bath. A 0.19% orcinol solution in 53% concentrated sulfuric acid, detectable spectrophotometrically at 421 nm, was used to dilute the samples by a factor of 10 in each.

### 2.4. Standard Curve for L-Rhamnose by Orcinol Reagent

The rhamnolipid concentration was calculated using the L-rhamnose (0–50 μg mL^−1^) standard curve, which was then represented as rhamnose equivalents. When rhamnose is used to generate the calibration curve, a correction factor must be applied to account for the higher mass of the lipidic component of rhamnolipids. Déziel et al. [55] calculated 2.25 as the adjustment factor.

### 2.5. LasR, RhlR, and QscR Reporter In Vitro Assays

Using a β-galactosidase reporter, we tested the in vitro activity of LasR, RhlR, and QscR in *E. coli* DH5, as previously described [56]. In brief, we grew a single colony of *E. coli* DH5 in LB medium with the plasmids pSC11 (lacZ reporter plasmid for LasR, QscR, or RhlR; see Table S1) [57,58] and pJN105 (LasR, QscR, and RhlR production plasmid; see Table S1) [59,60]. Overnight, the culture was diluted 1:10 into fresh LB medium containing 100 μg/mL ampicillin and 10 μg/mL gentamicin. A final concentration of 4 mg/mL of arabinose was applied after the cells had fully developed. A 2 μL aliquot of NBD stock solution (in DMSO) or DMSO alone (vehicle control) was added to each well of a 96-well microtiter plate. The full assay procedures are described in the supplementary file.

### 2.6. Production and Purification of LasR Protein

Full-length LasR (cloned into pET23b) was produced in BL21 *E. coli* cells using 1 mM IPTG, at 18 °C, overnight, in the presence of 100 μM OdDHL (for LasR-odDHL) or 100 μM NBD (for NBD-LasR). Cells were pelleted at 3000 rpm before being resuspended in lysis buffer (500 mM NaCl, 20 mM Tris-HCl, pH 8, 20 mM imidazole, 1 mM EDTA, 1 mM DTT, 5% glycerol). The soluble fraction was separated by centrifugation at 32,000× *g*. By diluting the soluble fraction fivefold in buffer A, the protein was prepared for heparin column binding. The protein was loaded onto a GE Healthcare heparin column and eluted using a linear gradient from buffer A to buffer B. SDS-PAGE was used to collect and analyze the peak fractions. The fractions were pooled and diluted five times in buffer A before being loaded into a MonoQ column (GE Healthcare) and eluted with a linear gradient from buffer A to buffer B. The materials were processed through size exclusion chromatography on a GE Healthcare S200 column in 20% buffer B after collecting peak fractions, pooling them, and concentrating them. The peak fractions were all mixed, concentrated to 2 mg/mL, flash-frozen, and kept at −280 °C.

### 2.7. Sedimentation Velocity Analysis

Sedimentation velocities were measured in quartz cells with double-sector centerpieces using a ProteomeLab XL-A analytical ultracentrifuge (Beckman Coulter^TM^, Brea, CA, USA) at 141,900× *g* and 20 °C. Absorption measurements were recorded at 280 nm at regular intervals of 150 s until the borders reached the cell’s base. Before centrifugation, the LasR samples were extensively dialyzed in 20 mM Hepes (pH 7.5) and 100 mM NaCl. In the tests, protein doses of 0.5 and 1 mg/mL were employed in the presence of either 100 μM OdDHL or 100 μM NBD. The solvent’s density and viscosity were 1.0004 and 0.0103, respectively. The data was analyzed using the SEDFIT [48] program’s continuous c(S) and continuous c(M) distributions. More information is available in the supplementary file (Table S3).

### 2.8. Thermal Shift Assay

The tested protein (i.e., LasR-odDHL or LasR-NBD) was diluted to 5 μM in a final volume of 20 μL of thermal shift assay reaction solution containing DMSO. A total of 2 μL of 200X SYPRO Orange in DMSO was added to the reaction buffer. The melting curve setting and fluorescence measurement with the ROX reporter setting on a QuantStudio 6 Flex PCR System (Applied Biosystems, Foster City, CA, USA) were used to analyze heat changes in 20 μL samples in 384-well plates. Following a two-minute soak at room temperature (25 °C), samples were heated linearly at 0.05 °C per second to 99 °C and held there for another two minutes.

### 2.9. In Silico Study

#### 2.9.1. Molecular Docking

Docking studies were performed using the AutoDock Vina software 1.3.2 [61] on a 3D model of LasR available in the Protein Data Bank (PDB code: 6V7W). The generated poses were analyzed using Pymol software (version 3.2.2) [62]. The procedure is detailed in the supplementary file.

#### 2.9.2. Molecular Dynamic Simulation

All molecular dynamics (MD) simulation studies were carried out using the Desmond software 12.2.1 [63]. The procedures are detailed in the supplementary file.

#### 2.9.3. Absolute Binding Free Energy Calculation

The absolute binding free energy (Δ*G*_binding_) was calculated using the Free Energy Perturbation (FEP) method [64]. The molecular dynamics simulation experiments required for this estimation were carried out using the NAMD 3.0.0 software [65,66]. The supplementary file has a full description of the procedure.

## 3. Results

In our continuous effort to find potent antibiofilm natural products that work against *P. aeruginosa*, we have found that one of our in-house crude extracts (i.e., *P. tinctorum* extract) exhibits interesting antibiofilm inhibition (41% biofilm inhibition at the subinhibitory concentration 50 μg/mL). Hence, this extract was selected for chromatographic isolation to purify the main bioactive compounds. Among the nine isolated compounds (Table S1), norlobaridone (NBD) showed significant antibiofilm activity against *P. aeruginosa*, which was higher than that of the reference inhibitor used, azithromycin (AZM) (64.6% and 52.7%, respectively), at the subinhibitory concentration of 5 μg/mL (Figure S2). At this dose, NBD had no effect on the growth of *P. aeruginosa*, as shown by the fact that the growth curve of the bacteria in the presence of 5 μg/mL NBD was identical to the curve in the absence of the treatment (Figure S1).

### 3.1. NBD Exhibits Selectivity for LasR in P. aeruginosa

**Figure 4:**
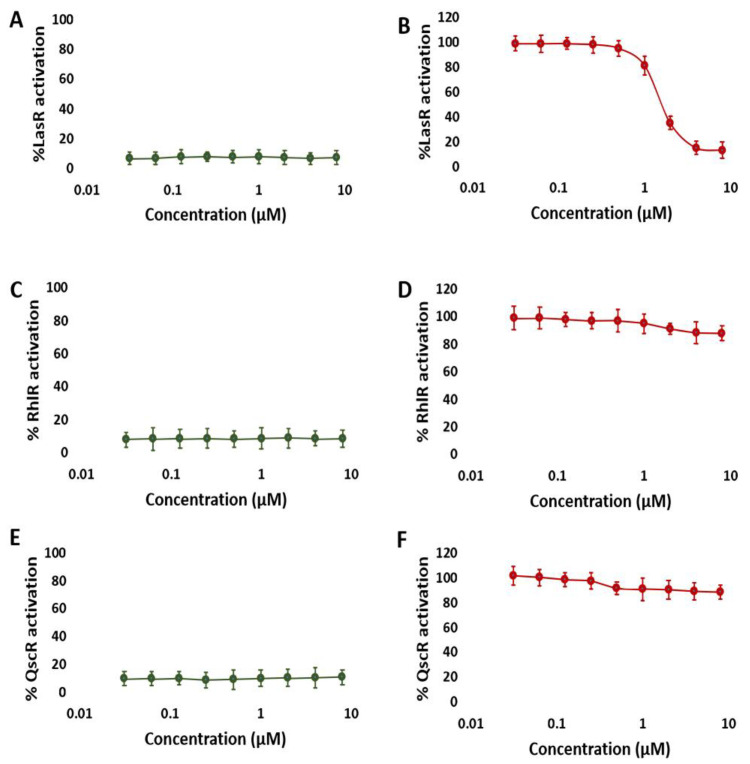
NBD dose-response β-galactosidase reporter assay curves in the LasR, RhlR, and QscR. NBD agonism (left; (**A**,**C**,**E**)) and antagonism (right; (**B**,**D**,**F**)) profiles in the LasR (**A**,**B**), RhlR (**C**,**D**), and QscR (**E**,**F**) reporters. These results suggest that NBD is a specific LasR antagonist. The data is plotted for at least three biological replicates, each of which has three technical replicates. The error bars represent standard deviation.

Our first objective was to determine if NBD might inhibit LuxR-type receptors (LasR, RhlR, and QscR) in *P. aeruginosa*. Using reporter gene assays (i.e., β-galactosidase reporter assay) in *E. coli*, we investigated the antagonistic and agonistic effects of NBD on LasR, RhlR, and QscR (see Methods for details). NBD’s antagonistic activity was evaluated against the native agonists of the receptors (i.e., odDHL and BHL), while NBD’s agonistic activity was evaluated independently. These reporter assays from *E. coli* allowed for a more detailed determination of selectivity profiles, where the ability to produce β-galactosidase indicated the degree of receptor activity. NBD only demonstrated significant dose-dependent antagonist activity against LasR (IC50 = 1.93 ± 0.21 μM; Figure 4B) and weak antagonistic activities against RhlR and QscR (IC50 = 49.61 ± 0.39 and 55.76 ± 0.45 μM, respectively). Regarding the agonistic activity, NBD was inactive toward the three tested receptors. As a result of these observations, NBD can be regarded as a promising LasR-selective competitive inhibitor.

### 3.2. NBD Interferes with LasR Dimerization

To further understand how NBD causes receptor antagonism, we investigated their interactions with LasR. Because LuxR-type receptors are unstable in vitro, even in the presence of their native AHL ligand, little is known about the mechanisms underlying the small-molecule antagonism of these receptors [67]. LasR antagonists have been shown to work by either: (i) destabilizing the receptor’s functional structure; (ii) blocking the direct interaction with DNA [68,69,70,71]; or by generating soluble complexes that are unable to dimerize into a functioning quaternary structure (i.e., LasR dimer) [70,71]; or possibly by a combination of these methods. Most previously described LasR competitive inhibitors (Figure 3C) were suggested to inhibit the receptor’s essential dimerization, rendering it unable to bind to DNA and promote gene expression [72,73,74].

**Figure 5:**
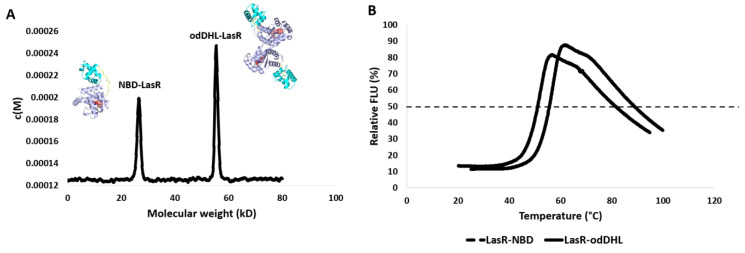
(**A**) LasR sedimentation velocity analysis. The c(M) size distribution function was used to fit the results. LasR had two different molecular weights depending on the ligand it was bound to. The binding of odDHL (the native LasR agonist) caused LasR to dimerize and have a molecular weight of 53.24 kD. In contrast, NBD binding prevents LasR dimerization, allowing it to appear at its true molecular weight of 27.58 kD. (**B**) The thermal shift assay for LasR shows that its complex with NBD (i.e., Lasr-NBD) has a lower Tm by 9.74 °C than its complex with the native ligand odDHL (i.e., LasR-odDHL).

Accordingly, we produced and purified full-length LasR in the presence of either its native agonist odDHL or NBD to see if NBD can disrupt LasR dimerization. The molecular weights of the resulting LasRs were then calculated using sedimentation velocity analysis. As shown in Figure 5A and Table S3, the molecular weight of LasR produced in the presence of NBD (i.e., NBD-LasR) was half that produced in the presence of the native autoinducer odDHL (27.58 kD and 55.36 kD, respectively). These results are quite close to the predicted molecular weights of single non-dimerized LasR (26.62 kD) and dimerized LasR (53.24 kD, respectively). As a result, these findings clearly show that NBD antagonizes LasR by interrupting its dimerization, which is required for it to bind to the bacterial DNA.

### 3.3. NBD Induces Destabilization of LasR

odDHL’s stabilizing action is a key factor in LasR dimerization into a functional complex; hence, the loss of this crucial dimerization step may be due to NBD’s destabilizing effect. NBD’s binding effect with LasR was determined in terms of the change in LasR’s melting temperature (ΔTm) in order to confirm this assumption.

As shown in Figure 5, LasR in complex with NBD had a considerably lower melting temperature than LasR in complex with the native ligand odDHL (Tm = 44.21 ± 0.38 °C and 53.95 ± 0.21 °C, respectively) with a ΔTm of 9.74 °C. Taken together, these results allow us to conclude that NBD interacts with LasR (presumably inside the odDHL binding pocket), rendering it substantially less stable than odDHL binding, and enabling it to oligomerize into its functional dimeric form.

### 3.4. NBD Reduced the Production of P. aeruginosa Virulence Factors

The *P. aeruginosa* pyocyanin and rhamnolipids production (i.e., QS-regulated virulence component) was also tested in the presence of a subinhibitory concentration of NBD (5 μg/mL). Compared to the reference inhibitor AZM (% of inhibition = 64.6% and 57.7%, respectively), NBD strongly inhibited pyocyanin and rhamnolipids (% of inhibition = 61.1% and 55%, respectively) (Figures S3 and S4).

Taken together, all of the in vitro results showed that NBD is a promising powerful antibiofilm drug against *P. aeruginosa* that selectively inhibits its LasR.

### 3.5. In Silico Study

#### 3.5.1. NBD Binds Similarly to odDHL Inside the LasR Ligand Binding Site

**Figure 6:**
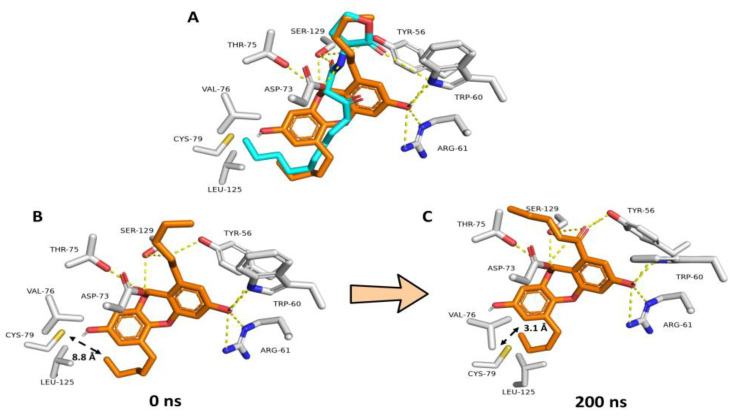
(**A**) Docking-based binding mode of NBD (orange structure) in alignment with odDHL (cyan structure) inside the LasR–odDHL binding pocket. (**B**,**C**) Dynamic binding modes of NBD over the course of 200 ns long MD simulation. Over the course of the simulation, the odDHL binding pocket shrunk leading to make VAL-76, CYS-79 and LEU-125 residues more close and able to form hydrophobic interactions with the terminal NBD’s alkyl arm.

NBD’s modeled structure was prepared and docked into the LasR ligand binding site to study its likely binding mechanism. All of the obtained docking poses had nearly identical orientations (Figure S5B). The modeled structure of the co-crystalized native ligand (i.e., odDHL) was also docked into the LasR ligand binding site to validate the docking methodology. The retrieved findings also had convergent orientations, and the best-scoring pose was precisely aligned with the co-crystalized one with an RMSD of 1.25 (Figure 6A).

Consequently, we used the NBD’s best scoring pose for the subsequent absolute binding free energy calculations (ΔGBind) and MD simulations. When the best docking poses of NBD and odDHL were aligned (Figure 6A), they demonstrated comparable binding modes. TYR-56, TRP-60, ASP-73, and SER-129 established H-bonds with both structures, whereas NBD formed two more H-bonds with ARG-61 and THR-75. Regarding hydrophobic interactions, both structures shared almost the same interactions, in which NBD demonstrated additional π-stacking interactions with TYR-56, while odDHL’s extended terminal alkyl moiety interacted with VAL-76, CYS-79, and LEU-125. Hydrophobic interactions with the latter three amino acid residues (i.e., VAL-76, CYS-79, and LEU-125) are required for LasR ligands to operate as agonists, according to our previously described LasR association–dissociation model. As a result, NBD, which had no hydrophobic interactions with the three amino acid residues, will most likely cause LasR-complex breakup during MD simulation. Furthermore, NBD’s overall molecular size is smaller than that of odDHL, which, according to our previously described model, makes the LasR complex dissociation assumption more feasible.

To study the stability of NBD and odDHL interactions inside the ligand binding site of LasR, the best-scoring binding pose for each structure was subjected to 200 ns long MD simulations using a single LasR subunit. The dynamic binding behaviors of NBD and odDHL were comparable to the static binding modes (Figure 6A). Furthermore, the computed ΔGBind values for each structure were convergent (−9.47 and −9.51 kcal/mol, respectively). An interesting observation extracted from the MD simulation of NBD-LasR was the significant shrinkage of the LasR ligand-binding pocket over the course of the simulations. The initial volume of the LasR ligand-binding site was calculated to be 1039 Å3. This volume shrunk to reach 855 Å3 at the end of the simulation. Accordingly, and because of the binding pocked shrinkage, VAL-76, CYS-79, and LEU-125 became closer to being able to interact with the terminal alkyl arm of NBD (Figure 6B,C). These findings are in great accordance with our previously reported ones about LasR inhibitors [72], where we found that this binding pocket shrinkage was the key step leading to the dissociation of the LasR dimeric form.

#### 3.5.2. NBD Destabilizes LasR

It also can be proposed that unstable LasR will be less likely able to form a stable functional dimer. Accordingly, from the aforementioned 200 ns long MD simulations we extracted the RMSD profiles of LasR as an expression of how LasR structure was stable over the course of the simulations.

**Figure 7:**
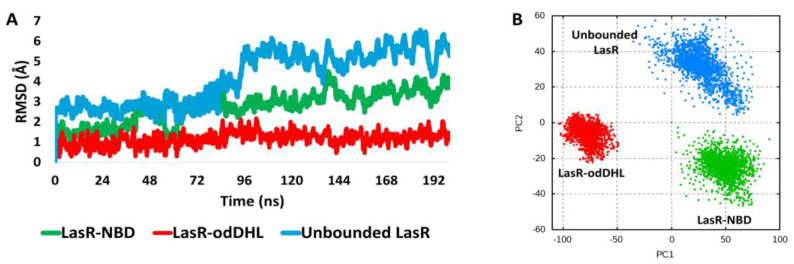
(**A**): RMSDs of LasR (single subunit) in complex with either NBD or the native ligand odDHL along with that of the free unbounded LasR over the course of 200 ns MD simulation. The presented results are the average values of three independent simulations. (**B**) Two-dimensional principal component analysis (PCA) projections of MD simulation trajectories.

As shown in Figure 7, the stability of the NBD–LasR complex (i.e., the RMSD profile during the course of the MD simulation) was clearly different from that of the odDHL–LasR complex. Over the course of simulation, the NBD–LasR complex exhibits a widely changing RMSD profile, with an average RMSD of 3.1 ± 0.71 (n = 3). This profile is comparable to the unliganded LasR profile (average RMSD = 4.4 ± 0.52). The odHDL–LasR complex, on the other hand, was substantially more stable, with a smaller fluctuating RMSD profile (average RMSD = 1.3 ± 0.31). These findings are consistent with the melting temperature assay (Figure 5B), indicating that NBD destabilizes LasR upon binding. Accordingly, upon unsupervised clustering of the LasR global structure movements using principal component analysis of the structure eigenvectors generated over the course of the MD simulation, the LasR’s three states (i.e., NBD-LasR, odDHL-LasR, and the unliganded LasR) were clearly separated from each other indicating that the state of the odDHL-binding pocket, in terms of its occupation with agonists (e.g., odDHL) or antagonists (e.g., NBD), or even if it was completely unoccupied, is reflected in the state of LasR’s whole structure.

#### 3.5.3. NBD Induces LasR Complex Dissociation upon MD Simulation

**Figure 8:**
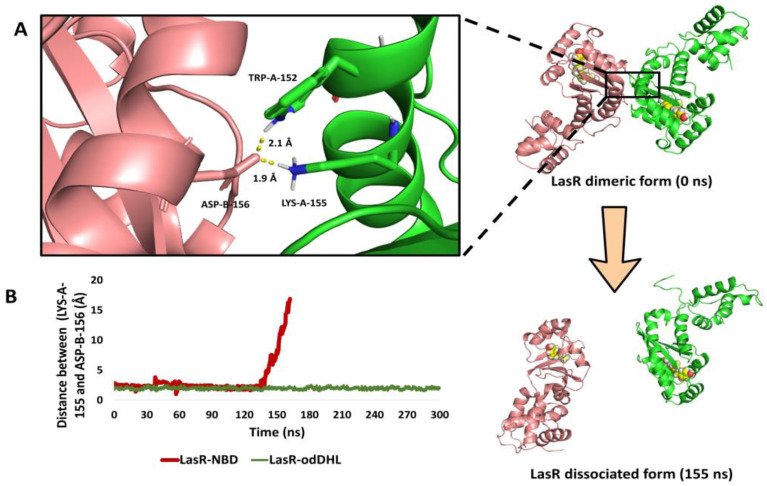
(**A**) The structure of the LasR dimeric form at 0 ns and after the dissociation (at 155 ns). The binding interface between the two subunits showed the key interacting amino acid residues (TRP-152, LYS-153, and ASP-156). (**B**) The calculated distance between LasR’s two subunits over the course of 300 ns long MD simulation (calculated as the distance between LYS-155 of one subunit and ASP-156 of the other one) in the presence of either NBD or odDHL.

As observed in the sedimentation velocity assay, binding of NBD with LasR inhibited its natural oligomerization into its functioning dimeric form (Figure 8). To study how NBD can cause LasR complex dissociation, the modelled LasR in its dimeric form bound to NBD was exposed to a 300 ns MD simulation. Following a careful investigation of the MD simulation findings, it was possible to make the following conclusions:

(i) In the presence of NBD, the ligand-binding pocket shrunk from 1039 Å3 to 844 Å3. This finding is consistent with that of the aforementioned 200 ns simulation of a single LasR subunit (Figure 8A,B) and with our recent results [72], in which the ligand-binding pocket decreased to 826 Å3 in the presence of a LasR antagonist.

(ii) As with previously reported inhibitors [72], the shrinking of the ligand-binding pocket led in an allosteric inward shift of ASN-136 to the ALA-166-helix (Figure S6). This induced inward shift promoted the dissociation of the LasR dimeric complex because this helix (ASN-136 to ALA-166-helix) contains the key interacting amino acid residues (TRP-152, LYS-153, and ASP-156; Figure 8A) that are involved in the H-bonding between LasR subunits to form a functioning LasR dimer [72]. At 142 ns, the dissociation event began, and the full dissociation was detected at 155 ns (Figure 8B).

## 4. Discussion

The lack of chemical probes for one of the primary QS receptors, LasR, in the opportunistic pathogen *P. aeruginosa* inspired our current investigation. There has been a lot of work conducted into discovering antagonists for LasR in *P. aeruginosa*, but they have not been very successful so far.

V-06-018, 4-bromo PHL, and their derivatives (Figure 3) are among the few well-characterized LasR antagonists reported in the literature [56,70], hence NBD can be considered a rare and valuable scaffold for further development and optimization. Further exploration into NBD’s mode of action revealed that this small organic molecule competes for the binding site with the native LasR agonist (odDHL).

Our findings showed that the binding of NBD inside the LasR ligand-binding pocket prevented the latter’s self-association into the functional dimer, and that this action is a direct result of NBD-induced LasR destabilization. Previous reports have shown that LasR can be inhibited via various mechanisms, including: (i) inhibition of LasR folding into its soluble functioning form (e.g., V-06-018; Figure 3); (ii) impairment of LasR binding with DNA (e.g., flavonoids); and (iii) indirect LasR downregulation (e.g., diallyl disulfide). Interestingly, in this study, we were able to simulate the dimeric LasR dissociation upon NBD binding and show that LasR in complex with NBD is substantially more unstable than the native ligand odDHL [70,73,75,76].

According to the modelling results, NBD binds similarly to odDHL inside the ligand binding pocket. However, this interaction does not involve hydrophobic interactions with VAL-76, CYS-79, or LEU-125. Hydrophobic interactions with VAL-76, CYS-79, and LEU-125 are essential for LasR ligands to act as agonists, according to our previously published LasR association–dissociation model [72]. As a result, NBD is likely to trigger LasR complex dissociation during MD simulation since it did not make any hydrophobic interactions with these three amino acid residues. We believe that the new structural and biochemical findings presented herein shed some light into the mechanism of the previously reported antibiofilm activity of Parmotrema lichens [46,47], and will aid in the development of more potent LasR modulators in the future.

## 5. Conclusions

In conclusion, our investigation identified NBD, for the first time, as a highly potent and specific LasR antagonist that should be widely used as a chemical probe in QS of *P. aeruginosa*, offering new insights into LasR antagonism processes. The new findings presented herein shed light on the cryptic world of the LuxR-type system as a key modulator of QS in this important and opportunistic pathogen.

## Data Availability

The data presented in this study are available in this article and supplementary material.

## Supplementary Materials

The following supporting information can be downloaded at: https://www.mdpi.com/article/10.3390/biom13111573/s1, Figures S1–S4: Antibiofilm activity; Figures S5–S6: Confirmational change of LasR; Figures S7–S9: NMR data; Table S1: Isolated compounds; Table S2: Primers and vectors used in the study; Table S3: Sedimentation velocity assay.
