# Supplementary material for: Norlobaridone Inhibits Quorum Sensing-Dependent Biofilm Formation and Some Virulence Factors in Pseudomonas aeruginosa by Disrupting Its Transcriptional Activator Protein LasR Dimerization
Source: Biomolecules. 2023 Oct 24;13(11):1573. doi: 10.3390/biom13111573 (PMC10669572; doi:10.3390/biom13111573)
Supplement: Supplementary file 1 [file biomolecules-13-01573-s001.zip › biomolecules-2648201-supplementary.pdf]

Supplementary Information

Norlobaridone inhibits quorum sensing-dependent biofilm formation and some virulence factors in *Pseudomonas aeruginosa* by disrupting its transcriptional activator protein LasR dimerization

Raya Soltane[a], Ahlam Alasiri[a], Mostafa N. Taha[b], Rehab H. Abd El-Aleam[c], Kawthar Saad Alghamdi[d] Mosad A. Ghareeb[e], Doaa El-Ghareeb keshek[f,g], Susana M. Cardoso[h], Ahmed M. Sayed[i*]

[a] Department of Basic Sciences, Adham University College, Umm Al-Qura University, Makkah 21955, Saudi Arabia. rasoltan@uqu.edu.sa; ajasiri@uqu.edu.sa

[b] Microbiology and Immunology Department, Faculty of Pharmacy, Nahda University, Beni-Suef, Egypt. moustafa.nasr@nub.edu.eg

[c] Pharmaceutical Chemistry Department, Faculty of Pharmacy, Modern University for Technology and Information MTI, Cairo 11571, Egypt. Rehab.hamed@pharm.mti.edu.eg

[d] Department of Biology, College of Science, University of Hafr Al Batin, Hafar Al Batin, Saudi Arabia. Ksalghamdi@uhb.edu.sa

[e] Medicinal Chemistry Department, Theodor Bilharz Research Institute Kornaish El Nile, Warrak El-Hadar, Imbaba (P.O. 30), Giza 12411, Egypt. m.ghareeb@tbri.gov.eg

[f]Department of biology, Jumum College University, Umm Al-Qura University, Makkah 21955, Saudi Arabia.

[g]Agriculture Genetic Engineering Research Institute (AGERI), Agriculture Research Center, Giza, Egypt. dekeshek@uqu.edu.sa

[h]Associated Laboratory for Green Chemistry of the Network of Chemistry and Technology LAQV-REQUIMTE, Department of Chemistry, University of Aveiro, 3810-193 Aveiro, Portugal. susanacardoso@ua.pt

[i]Pharmacognosy Department, Faculty of Pharmacy, Nahda University, Beni Suef, Egypt. ahmed.mohamed.sayed@nub.edu.eg

* Corresponding author

E-mail addresses: ahmed.mohamed.sayed@nub.edu.eg (Ahmed M. Sayed); rasoltan@uqu.edu.sa (Raya Soltane)

Table S1. Structures, antibacterial (in µg/mL) and antibiofilm (in % of inhibition) activities of the compounds isolated from *P. tinctorum* extract.

No.	Compound	Structure	% *P. aeruginosa* biofilm inhibition*	*P. aeruginosa* MIC	*E. coli* MIC
1	**Norlobaridone (NBD)**	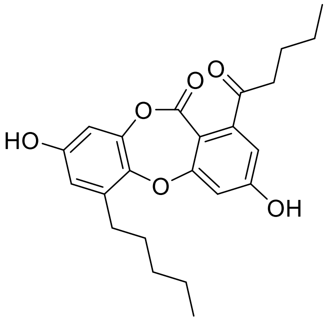	**<u>64.6</u>**	128	128
2	Pentylphenol	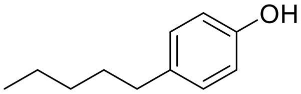	11.4	256	>256
3	Rottlerin	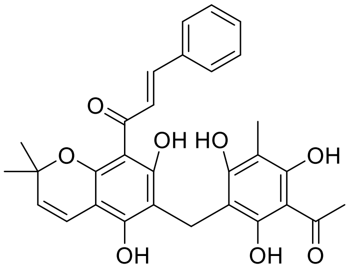	7.9	>256	>256
4	Ellagic acid	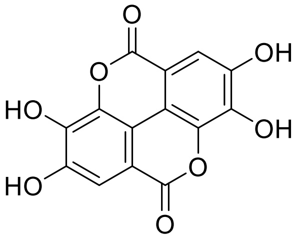	22.8	128	128
5	Gallic acid	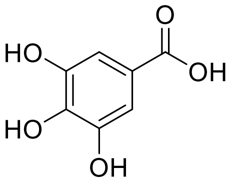	21.4	>256	>256
6	P-hydroxy benzoic acid 10	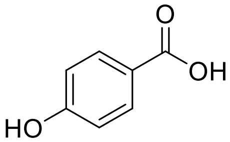	12.9	128	>256
7	Phloroglucinol	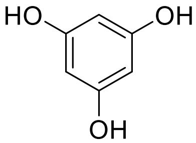	31.1	64	64
8	Resorcinol	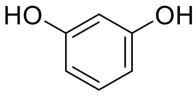	27.7	128	128

9	Acetophenone	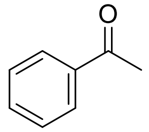	25.2	256	128
10	**AZM****	-	52.7	8	32
11	***P. tinctorum* extract**	-	44.5	64	128

*Antibiofilm activity expressed as the % of biofilm inhibition at the subinhibitory concentration (i.e., 5 µg/mL).

**AZM = Azithromycin, the reference antibiofilm drug.

Table S2. Bacterial strains and plasmids used in this study

Bacterial strain	Description	Reference
P. aeruginosa	-	ATCC27853
E. coli DH5α	Competent Cells	ThermoFisher (catalog number: 18265017)
BL21 (DE3) E. coli	Competent Cells	ThermoFisher (catalog number: EC0114)
**Plasmid**	**Description**	**Reference**
pSC11-L	*Broad host range* lasI'-lacZ *reporter; ApR*	Chugani et al. 2001
pSC11-Q	*Broad host range* PA1897'-lacZ *reporter; ApR*	Chugani et al. 2001
pSC11-R	*Broad host range* rhlI'-lacZ *reporter; ApR*	Chugani et al. 2001
pJN105L	*Arabinose-inducible* lasR *expression vector; pBBRMCS backbone; GmR*	Lee et al. 2006
pJN105Q	*Arabinose-inducible* qscR *expression vector; pBBRMCS backbone; GmR*	Lee et al. 2006
pJN105R	*Arabinose-inducible* rhlR *expression vector; pBBRMCS backbone; GmR*	Eibergen et al. 2015
pET23b.LasR	*Full length LasR overexpression vector*	Paczkowski et al. 2017

Table S3. Details of sedimentation velocity data analysis

Protein	$S_{20,w}$[c]	MW[d]
LasR-odDHL[a]	3.112	53304
LasR-NBD[b]	1.813	26635

[a]LasR (0.5 mg/mL) in the presence of 100 µM odDHL.

[b]LasR (0.5 mg/mL) in the presence of 100 µM NBD.

[c]S20,w is the sedimentation coefficient with the parameter being corrected to 20.0 °C and the density of water.

[d]Molecular weight in Daltons.

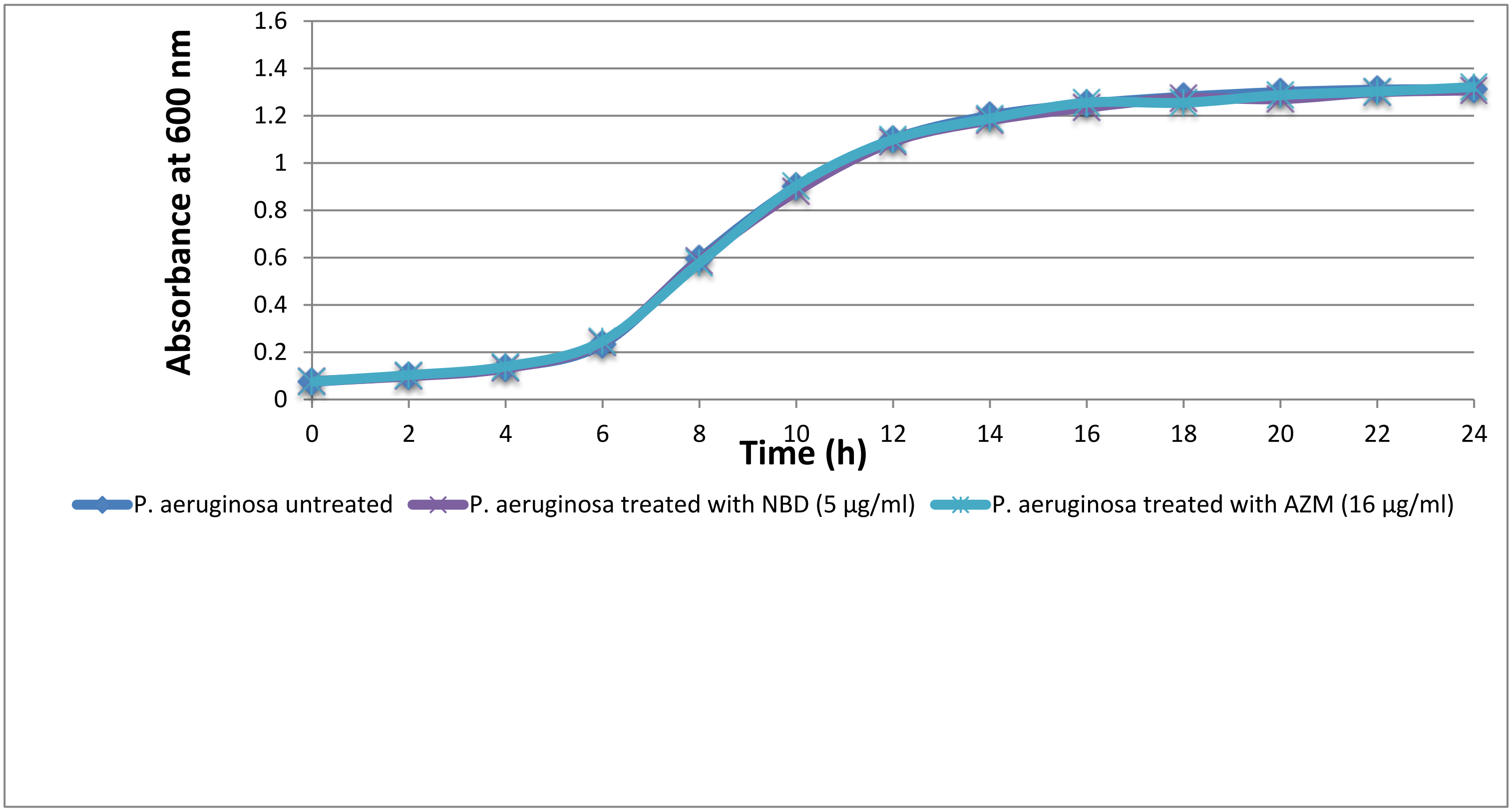

Figure S1. The effect of sub-MIC (5µg/mL) of NBD on *P. aeruginosa* growth at 37°C for 24 hr. The data represent the mean values of experiments performed in triplicate.

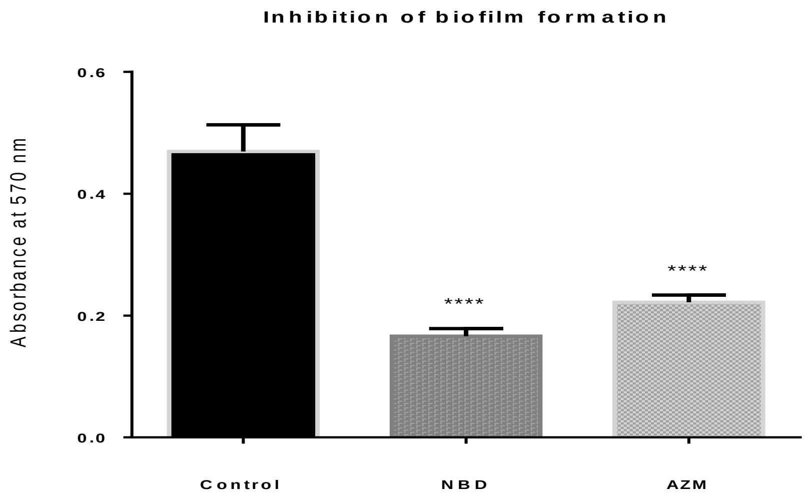

Figure S2. Inhibition of *P. aeruginosa* biofilm formation in the presence of sub-MIC of NBD and AZM. Data is an average of three replicates. The results are presented as the mean ± SD of the absorbance at 570 nm. * Statistically significant from the control (un-treated) at P<0.05.

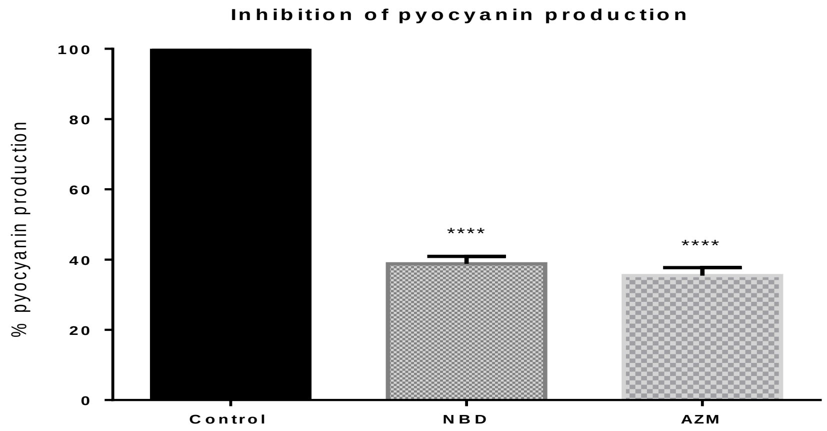

Figure S3. Inhibition of *P. aeruginosa* pyocyanin production in the presence of sub-MIC of NBD and AZM. Data is an average of three replicates. The results are presented as the mean ±SD of the percent of pyocyanin production. * Statistically significant from the control (un-treated) at P<0.05.

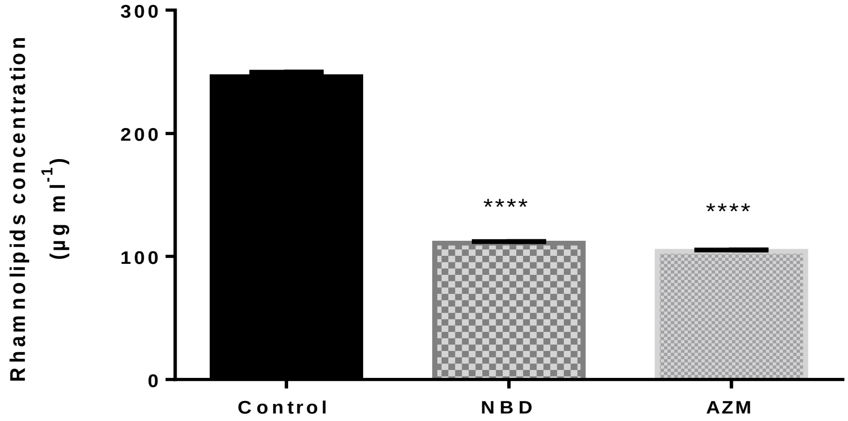

Figure S4. Inhibition of *P. aeruginosa* rhamnolipids concentration in the presence of sub-MIC of NBD and AZM. Data is an average of three replicates. The results are presented as the mean ± SD. * Statistically significant from the control (un-treated) at P<0.05.

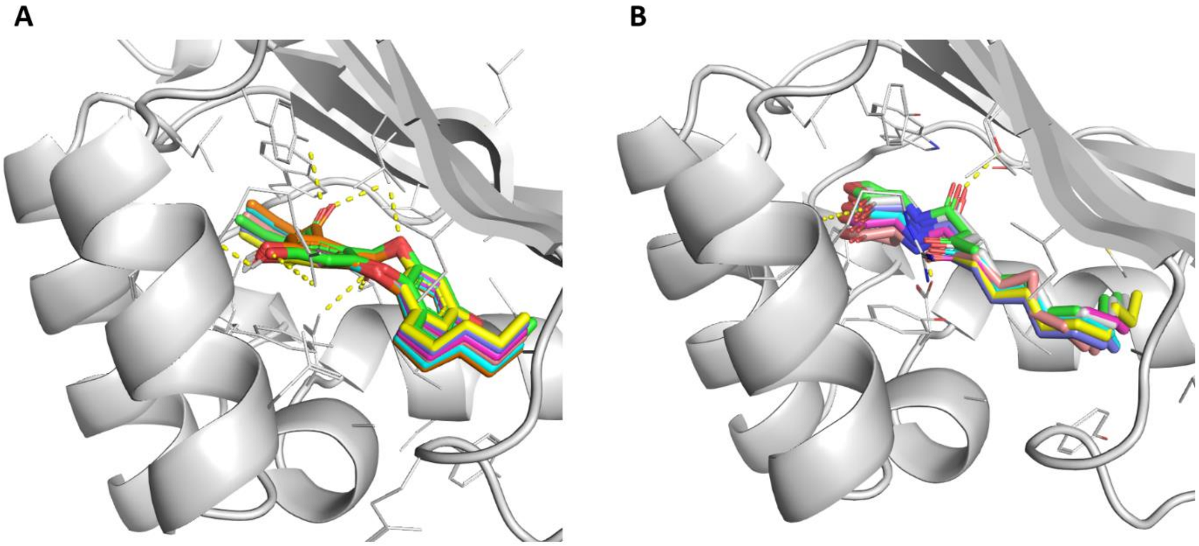

Figure S5. A: Docking poses of NBD in alignment with each other inside the LasR ligand binding domain. **B:** Docking poses of odDHL in alignment with the co-crystalized one (Yellow structure; RMSD ~ 1.254 Å).

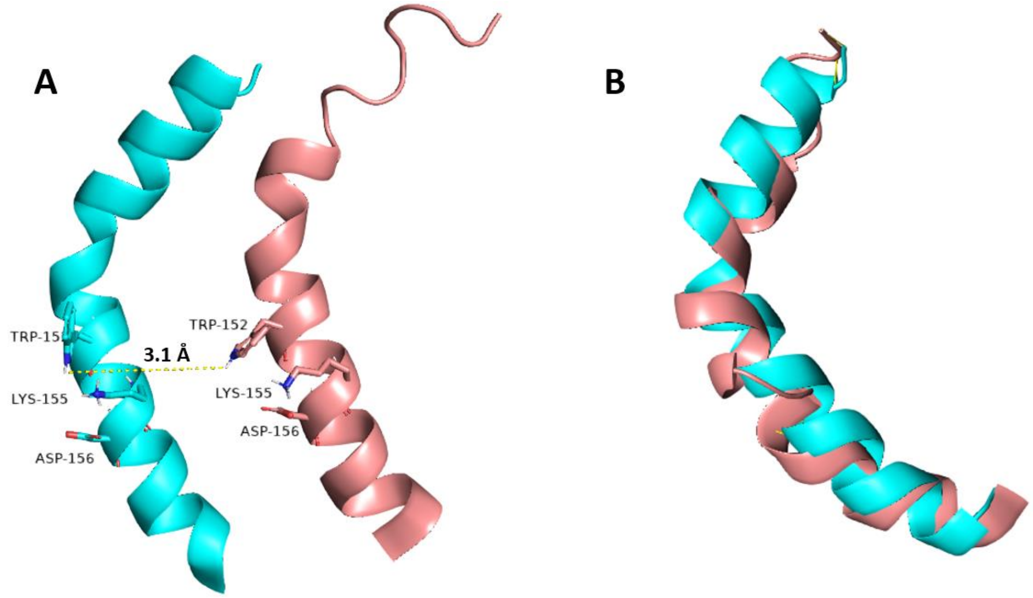

Figure S6. Inward movement of ASN-136 to ALA-166 α-helix of the Bak-bound LasR structures (A) over the course of 300ns long MDS. Blue-colored α-helix represent the native position at the beginning of MD simulation, while brick-red-colored α-helix represent the new position at the end of MD simulation. In case odDHL-bound LasR structures (B), ASN-136 to ALA-166 α-helix remained in its original position over the course of simulation. TRP-152, LYS-153, and ASP-156 are the key amino acid involved in the H-bonding between LasR subunits to form functioning LasR dimer.

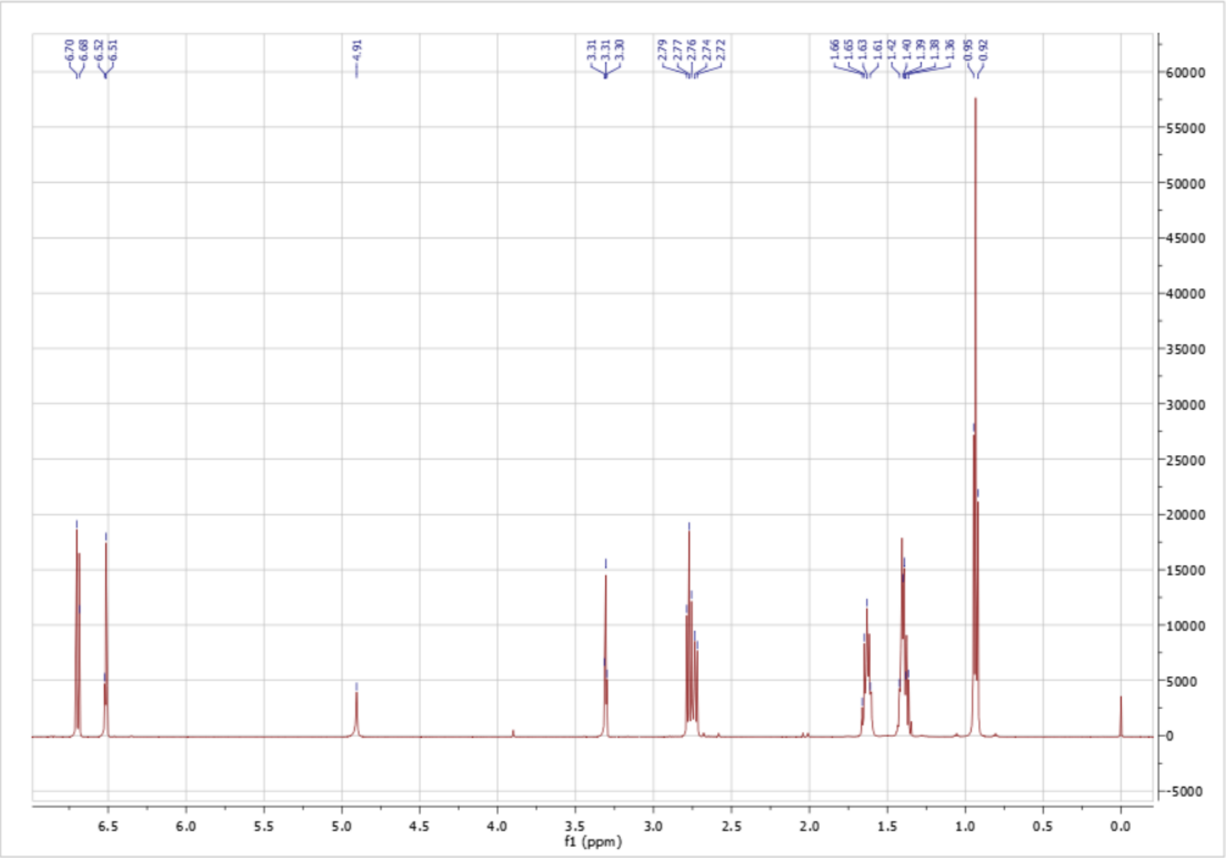

Figure S7. ^{1}H NMR spectrum of NBD in CD_3OD at 400 MHz.

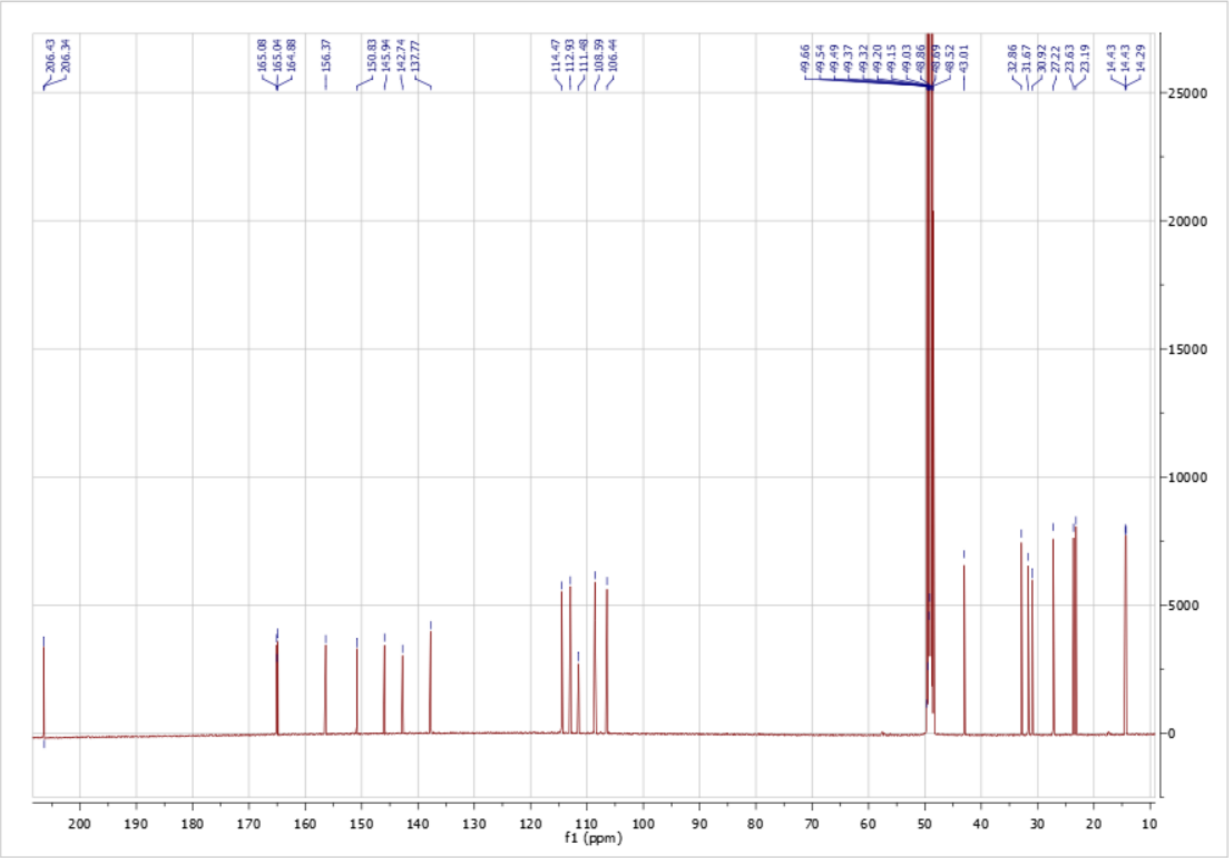

Figure S8. ^{13}C NMR spectrum of NBD in CD_3OD at 100 MHz.

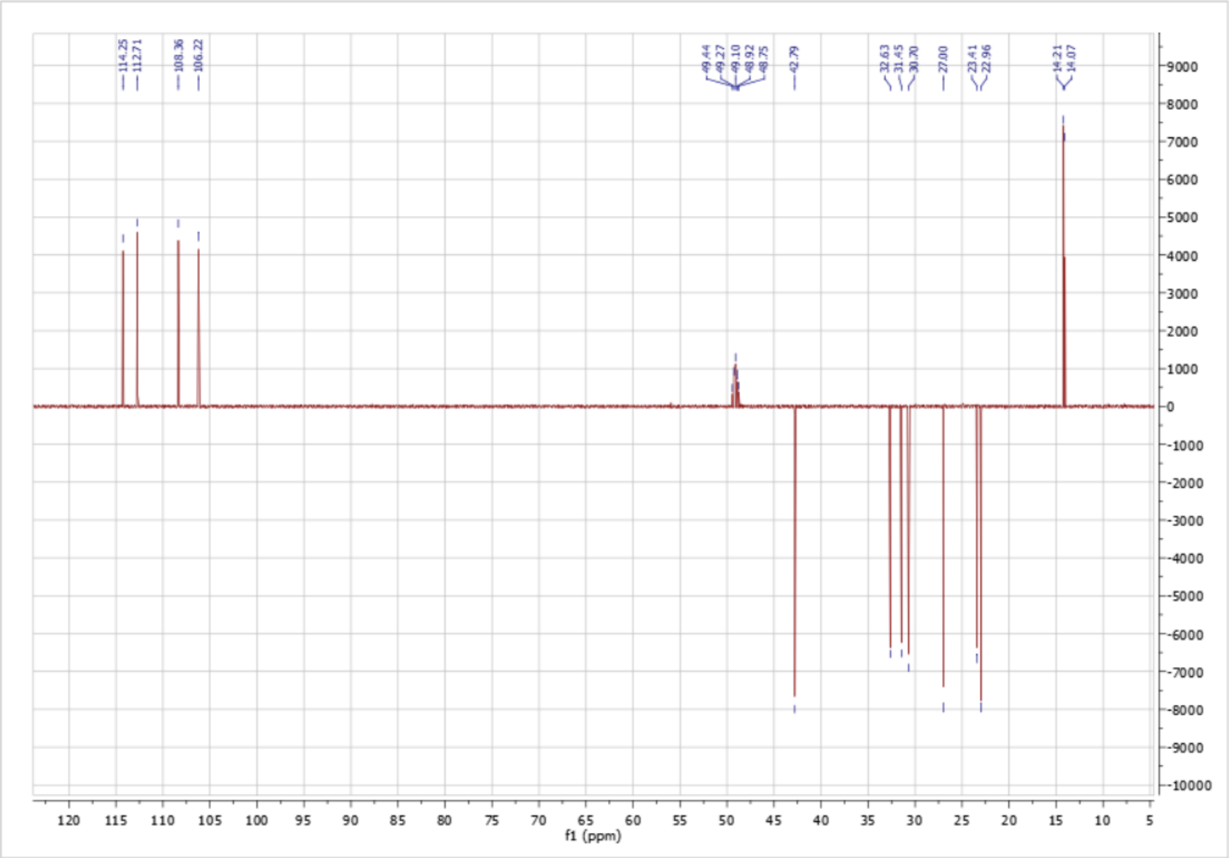

Figure S9. DEPT-135 NMR spectrum of NBD in CD_3OD at 100 MHz.

Methods

E. coli reporter β-galactosidase assays

LasR, QscR, and RhlR assays in E. coli αDH5 were carried out according to previously published techniques (Eibergen et al. 2015; O'Reilly and Blackwell 2016; Mattmann et al. 2011; Gerdt et al. 2015). Briefly, a single colony of E. coli strain αDH5 transformed with pSC11 (lacZ reporter plasmid for LasR, QscR, or RhlR; see Table S1) and pJN105 (LasR, QscR, and RhlR production plasmid) was inoculated in a 5-mL sample of LB medium containing 100 µg/mL Ampicillin and 10 µg/mL Gentamicin. The culture was cultivated overnight at 37 °C with 200 rpm shaking.

Overnight culture was diluted 1:10 in fresh LB medium with 100 µg/mL Ampicillin and 10 µg/mL Gentamicin and grown to an OD_{600} = 0.23-0.27. Aliquots of Bak stock solutions in DMSO (2 µL) were applied to the interior wells of a transparent 96-well microtiter plate, with final DMSO concentrations of 1%.

For antagonism experiments, once the subculture had achieved the desired OD, arabinose was added at a final concentration of 4 mg/mL to promote pJN105 expression.

As a negative control, six DMSO-containing wells were filled with 200 µL of this subculture. The remaining culture was adjusted to a final concentration of 2 nM OdDHL (for LasR), 10 nM OdDHL (for QscR), or 10 µM BHL (for RhlR), and 200 µL aliquots of treated culture were introduced to all experimental wells and DMSO wells (the latter as a positive control). The plate's outer wells were filled with 200 µL of water (to help maintain plate humidity and limit evaporation in the inside wells). For 4 hours, plates were incubated at 37 °C with shaking at 200 rpm.

For agonism experiments, 2 µL DMSO was added to six wells (negative control), and another 6 wells were treated with either 10 mM OdDHL in DMSO (for LasR and QscR) or 100 mM BHL (for RhlR). Once the subculture attained the desired OD_{600}, arabinose was added at a final dosage of 4 mg/mL to stimulate pJN105 expression. This culture was dispensed in aliquots (200 µL) into all internal wells. The plate's exterior wells were filled with 200 µL of water (to help maintain the plate's humidity/environment and delay overall evaporation for the internal wells). For 4 hours, plates were incubated at 37 °C with 200 rpm shaking.

The OD_{600} of each cell was measured after the period of incubation. Following that, 200 µL Z buffer, 8 µL $CHCl_3$, and 4 µL 0.1% aqueous SDS were added to each interior well of a new 96 well plate. Each well in the original plate had a 50-µL aliquot transferred to the lysis-buffer containing plate and lysed by aspirating 15 times. Following that, the receptor assays differed, as shown below.

Inregard to LasR assay, a 100-µL aliquot from each well was transferred to a new, clear-bottom 96-well plate. At t = 0 minutes, the Miller assay (measuring β-galactosidase enzyme activity) was begun by adding 20 µL of the substrate *ortho*-nitrophenyl-β-galactoside (ONPG, 4 mg/mL in phosphate buffer) to each well. The plates were then incubated at 30 °C without shaking for 30 minutes. The enzymatic reaction was stopped by adding 50 µl of sodium carbonate (1M in water)

to each well. Absorbances at 420 and 550 nm were measured for each well. The following formula was used to determine Miller units:

$$Miller\ unit = \frac{1000\ \times (A420 - (1.75\ \times A550))}{t\ \times\ \nu\ \times OD600}$$

t = time ONPG incubated with lysate in min and ν = volume of culture lysed in mL

Miller units were reported as a percentage of the OdDHL-only contained wells (i.e., wells containing just agonist = 100% activity). All experiments were performed in technical triplicate and at least three times.

Regarding the QscR and RhlR assays, a 150 μL aliquot from each well was transferred to a new, transparent bottom 96-well plate. At t = 0 minutes, the Miller assay was initiated by adding 25 μL of substrate chlorophenol red-β-galactopyranoside (CPRG, 4 mg/mL in phosphate buffered saline) and incubating at 30 °C for 20 minutes (for RhlR) or 45 minutes (for QscR). Miller units were calculated using the following formula:

$$Miller\ unit = \frac{1000\ \times (A570)}{t\ \times\ \nu\ \times OD600}$$

t = time CPRG incubated with lysate in min and ν = volume of culture lysed in mL

Miller units were given as a percentage of wells containing just OdDHL (for QscR) or BHL (for RhlR) (i.e., wells containing only agonist equaled 100% activity). All assays were carried out in technical triplicate and at least three times.

In silico investigation and modeling methods

Docking

AutoDock Vina software was used in all molecular docking experiments (Seeliger et al. 2010). All isolated compounds were docked against the LasR crystal structures (PDB codes: 6V7W and 3IX4*)*. The binding site was determined according to the enzyme's co-crystallized ligand. The co-ordinates of the docking grid box for the first LasR subunit were: x = -33.9; y = 15.6; z = -31.31; and for the second were: x = - 53.16; y = 9.36; z = -2.93. The size of the grid box was set to be 10 Å. Exhaustiveness was set to be 24. Ten poses were generated for each docking experiment. Docking poses were analyzed and visualized using Pymol software (Seeliger et al. 2010). To validated the docking protocol, the co-crystalized ligands (odDHL and TP1) were re-docked inside the LasR binding pocket, where the produced poses were almost identical to that of the original crystalized ones (Figures S53 and S54) with RMSDs of 1.25 Å and 1.19 Å, respectively (Figure S1).

Molecular Dynamics Simulation

Desmond v. 2.2 software was used for performing MDS experiments (Bowers et al. 2006). This software applies the OPLS force field. Protein systems were built using the System Builder option, where the protein structure was checked for any missing hydrogens, the protonation states of the amino acid residues were set (pH = 7.4), and the co-crystalized water molecules

were removed. Thereafter, the whole structure was embedded in an orthorhombic box of TIP3P water together with 0.15 M Na^+ and Cl^- ions in 20 Å^3 solvent buffer. Afterward, the prepared systems were energy minimized and equilibrated for 10 ns. For protein-ligand complexes, the top-scoring poses were used as a starting point for simulation. Desmond software automatically parameterizes inputted ligands during the system building step according to the OPLS force field. For $\Delta G_{\text{binding}}$ simulations performed by NAMD (Phillips et al. 2005), the protein structures were built and optimized by using the QwikMD toolkit of the VMD software. The parameters and topologies of the compounds were calculated using the Charmm27 force field with the online software Ligand Reader and Modeler (http://www.charmm-gui. org/?doc=input/ligandrm) (Kim et al. 2020). Afterward, the generated parameters and topology files were loaded to VMD to readily read the protein–ligand complexes without errors and then conduct the simulation step. All produced trajectories were analyzed by Maestro or VMD software.

Absolute binding Free energy calculation

Binding free energy calculations (ΔG) were performed using the free energy perturbation (FEP) method (Kim et al. 2020). This method was described in detail in the recent article by Kim and coworkers (Kim et al. 2020). Briefly, this method calculates the binding free energy $\Delta G_{\text{binding}}$ according to the following equation: $\Delta G_{\text{binding}} = \Delta G_{\text{Complex}} - \Delta G_{\text{Ligand}}$. The value of each ΔG is estimated from a separate simulation using NAMD software. All input files required for simulation by NAMD can be prepared by using the online website Charmm-GUI (https://charmm-gui.org/?doc=input/afes.abinding). Subsequently, we can use these files in NAMD to produce the required simulations using the FEP calculation function in NAMD. The equilibration (5 ns long) was achieved in the NPT ensemble at 300 K and 1 atm (1.01325 bar) with Langevin piston pressure (for “Complex” and “Ligand”) in the presence of the TIP3P water model. Then, 10 ns FEP simulations were performed for each compound, and the last 5 ns of the free energy values was measured for the final free energy values (Kim et al. 2020). Finally, the generated trajectories were visualized and analyzed using VMD software.

References

Bowers, K.J.; Chow, D.E.; Xu, H.; Dror, R.O.; Eastwood, M.P.; Gregersen, B.A.; Klepeis, J.L.; Kolossvary, I.; Moraes, M.A.; Sacerdoti, F.D.; et al. Scalable algorithms for molecular dynamics simulations on commodity clusters. In Proceedings of the SC’06: Proceedings of the 2006 ACM/IEEE Conference on Supercomputing, Tampa, FL, USA, 11–17 November 2006; IEEE: New York, NY, USA, 2006; p. 43.

Chugani, S. A.; Whiteley, M.; Lee, K. M.; D'Argenio, D.; Manoil, C.; Greenberg, E. P., QscR, a modulator of quorum-sensing signal synthesis and virulence in *Pseudomonas aeruginosa*. *Proc. Natl. Acad. Sci. U. S. A.* **2001,** *98* (5), 2752-7.

Eibergen, N. R.; Moore, J. D.; Mattmann, M. E.; Blackwell, H. E., Potent and Selective Modulation of the RhlR Quorum Sensing Receptor by Using Non-native Ligands: An Emerging Target for Virulence Control in *Pseudomonas aeruginosa*. *Chembiochem* **2015,** *16* (16), 2348-56.

Gerdt, J. P., McInnis, C. E., Schell, T. L., & Blackwell, H. E. (2015). Unraveling the contributions of hydrogen-bonding interactions to the activity of native and non-native ligands in the quorum-sensing receptor LasR. *Organic & biomolecular chemistry*, *13*(5), 1453-1462.

Kim, S.; Oshima, H.; Zhang, H.; Kern, N.R.; Re, S.; Lee, J.; Rous, B.; Sugita, Y.; Jiang, W.; Im, W. CHARMM-GUI free energy calculator for absolute and relative ligand solvation and binding free energy simulations. J. Chem. Theory Comput. 2020, 16, 7207–7218.

Lee, J. H.; Lequette, Y.; Greenberg, E. P., Activity of purified QscR, a *Pseudomonas aeruginosa* orphan quorum-sensing transcription factor. *Mol. Microbiol.* **2006,** *59* (2), 602-9.

Mattmann, M. E., Shipway, P. M., Heth, N. J., & Blackwell, H. E. (2011). Potent and Selective Synthetic Modulators of a Quorum Sensing Repressor in Pseudomonas aeruginosa Identified from Second-Generation Libraries of N-Acylated L-Homoserine Lactones. *ChemBioChem*, *12*(6), 942-949.

O'Reilly, M. C., & Blackwell, H. E. (2016). Structure-based design and biological evaluation of triphenyl scaffold-based hybrid compounds as hydrolytically stable modulators of a LuxR-type quorum sensing receptor. *ACS infectious diseases*, *2*(1), 32-38.

Paczkowski, J. E., Mukherjee, S., McCready, A. R., Cong, J. P., Aquino, C. J., Kim, H., ... & Bassler, B. L. (2017). Flavonoids suppress Pseudomonas aeruginosa virulence through allosteric inhibition of quorum-sensing receptors. *Journal of Biological Chemistry*, *292*(10), 4064-4076.

Phillips, J.C.; Braun, R.; Wang, W.; Gumbart, J.; Tajkhorshid, E.; Villa, E.; Chipot, C.; Skeel, R.D.; Kalé, L.; Schulten, K. Scalable molecular dynamics with NAMD. J. Comput. Chem. 2005, 26, 1781– 1802.

Seeliger, D.; de Groot, B.L. Ligand docking and binding site analysis with PyMOL and Autodock/Vina. J. Comput. Aided Mol. Des. 2010, 24, 417–422.
